# Establishment and validation of prognosis model for patients with cerebral contusion

**DOI:** 10.1186/s12883-021-02482-4

**Published:** 2021-11-29

**Authors:** Yufeng Zhu, Xiaoqing Jin, Lulu Xu, Pei Han, Shengwu Lin, Zhongsheng Lu

**Affiliations:** 1 Department of Graduate School, Qinghai University, Xining, 810016 Qinghai China; 2 Department of Neurosurgery, Qinghai Provincial People’s Hospital, Xining, 810007 Qinghai China; 3 Department of Geriatric Medicine, the Affiliated Hospital of Qingdao University, Qingdao, 266000 Shandong China

**Keywords:** Traumatic brain injury;cerebral contusion, Cerebral edema, Nomogram, Prognosis

## Abstract

**Background And Objective:**

Cerebral Contusion (CC) is one of the most serious injury types in patients with traumatic brain injury (TBI). In this study, the baseline data, imaging features and laboratory examinations of patients with CC were summarized and analyzed to develop and validate a prediction model of nomogram to evaluate the clinical outcomes of patients.

**Methods:**

A total of 426 patients with cerebral contusion (CC) admitted to the People’s Hospital of Qinghai Province and Affiliated Hospital of Qingdao University from January 2018 to January 2021 were included in this study, We randomly divided the cohort into a training cohort (*n* = 284) and a validation cohort (*n* = 142) with a ratio of 2:1.At Least absolute shrinkage and selection operator (Lasso) regression were used for screening high-risk factors affecting patient prognosis and development of the predictive model. The identification ability and clinical application value of the prediction model were analyzed through the analysis of receiver operating characteristic curve (ROC), calibration curve, and decision curve analysis (DCA).

**Results:**

Twelve independent prognostic factors, including age, Glasgow Coma Score (GCS), Basal cistern status, Midline shift (MLS), Third ventricle status, intracranial pressure (ICP) and CT grade of cerebral edema,etc., were selected by Lasso regression analysis and included in the nomogram. The model showed good predictive performance, with a C index of (0.87, 95% CI, 0.026–0.952) in the training cohort and (0.93, 95% CI, 0.032–0.965) in the validation cohort. Clinical decision curve analysis (DCA) also showed that the model brought high clinical benefits to patients.

**Conclusion:**

This study established a high accuracy of nomogram model to predict the prognosis of patients with CC, its low cost, easy to promote, is especially applicable in the acute environment, at the same time, CSF-glucose/lactate ratio(C-G/L), volume of contusion, and mean CT values of edema zone, which were included for the first time in this study, were independent predictors of poor prognosis in patients with CC. However, this model still has some limitations and deficiencies, which require large sample and multi-center prospective studies to verify and improve our results.

**Supplementary Information:**

The online version contains supplementary material available at 10.1186/s12883-021-02482-4.

## Introduction

Traumatic brain injury (TBI) is the leading cause of death and disability worldwide, second only to limb fractures, and has been referred to as the “silent epidemic”.However, hemorrhagic CC is one of the most serious types of TBI, occurring in 20–30% of TBI patients [1]. CC increases the risk of disability and death in patients with TBI, causing serious negative impact on the daily life of patients and caregivers, and also causing long-term social and economic burden on families and society [2]. So far, the proposed of TBI classification based on computed tomography (CT) in the system is still in Marshall’s classification of the highest recognition, Marshall classification for the prognosis of TBI offers a wide range of information, including compression of basal cistern, midline shift (MLS) and the volume of contusion, etc., and is known for showing a good correlation with the results [3]. However, a major limitation of existing CT classifications is that most of them are based on CT scan morphological features (basilar cisterna status, MLS, intracerebral hemorrhage, punctate hemorrhage, etc.) to assess the patient’s condition and prognosis, and these morphological features are susceptible to factors such as physician level and progression of the disease. In addition, it is not comprehensive to evaluate the prognosis of patients only from the perspective of imaging. However, there are still few neurosurgical studies on the prognostic value of patients with CC. Therefore, this study attempted to screen out independent predictors of poor prognosis of patients with CC according to the baseline information, imaging and laboratory test results of patients with CC, and establish a Normogram model to evaluate the prognosis of patients and verify it.

### Patients

**Fig. 1:**
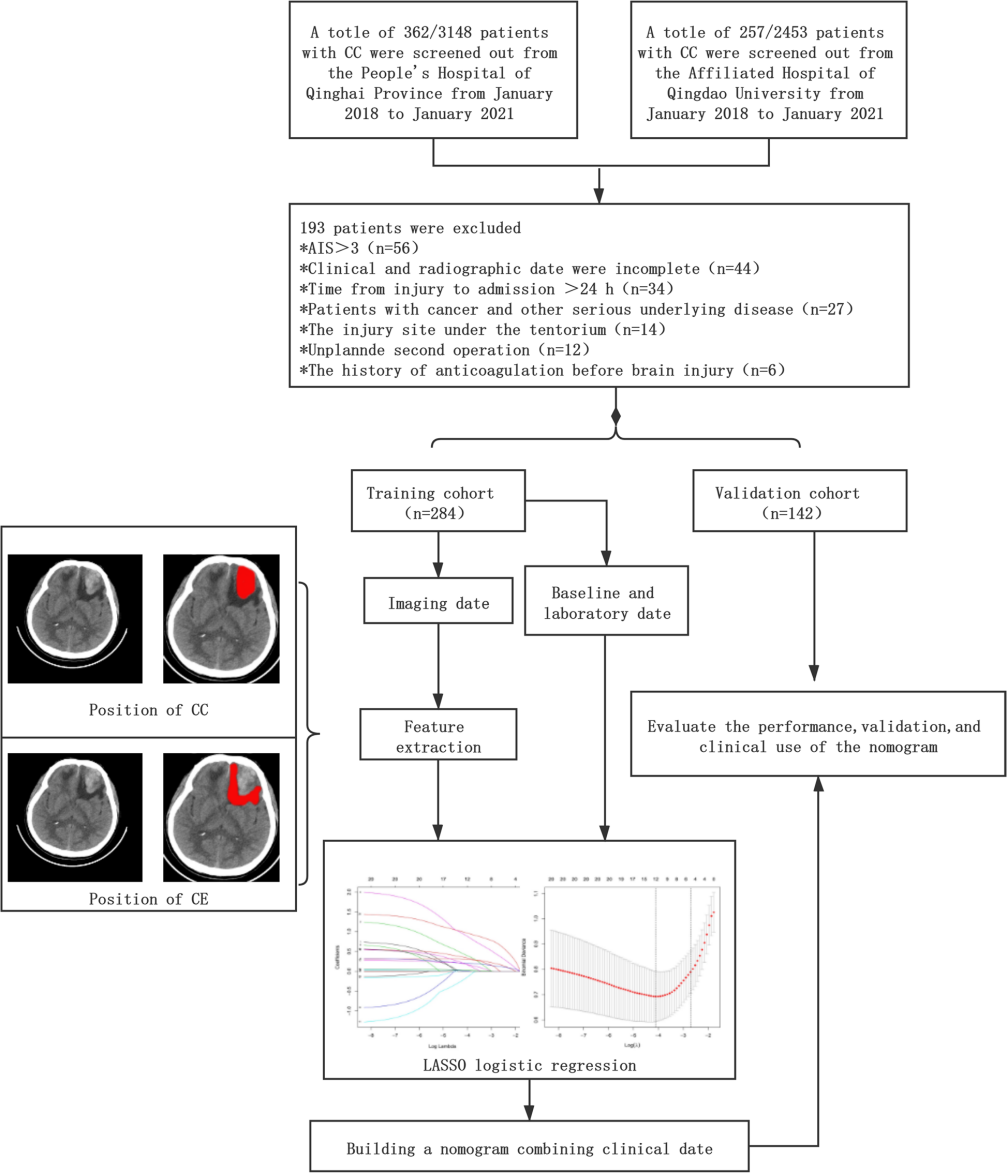
Study group and process flow chart. Patients with cerebral contusion were divided into two different groups (training group and validation group), and the data for statistical analysis were obtained from the training group

Clinical data of all patients with CC admitted to the People’s Hospital of Qinghai Province and Affiliated Hospital of Qingdao University from January 2018 to January 2021 were retrospectively analyzed, including patients’ baseline information, laboratory and imaging examinations. As shown in Fig. 1, 619 patients with CC were selected from the 5601 patients with TBI, and 426 patients finally met the inclusion criteria. We excluded 193 patients for the following reasons: 56 patients had a abbreviated injury scale (AIS) other than the head ≥3, and 44 patients had incomplete clinical and radiographic data.34 patients from injury to admission time > 24 h,27 patients had previous cerebral infarction and other serious underlying diseases, 14 patients had the injury site under the tentorium, 12 patients had the unplanned second operation, and 6 patients had the history of anticoagulation before brain injury.

Inclusion Criteria:

- ①. > 18 years old
- ②.CC diagnosed by head CT was supratentorial and AIS other than the head< 3 points
- ③.The first CT time after trauma was less than 24 h, and the CT images were reexamined on the third day of hospitalization
- ④. Complete GCS data at admission and 3-month follow-up after discharge
- ⑤.All TBI patients were treated in strict accordance with the clinical guidelines of neurosurgery

Exclusion Criteria:

- ①Children, pregnant women and people with coagulation dysfunction or who are currently receiving anticoagulant therapy
- ②.Previous cerebral infarction and intracranial space occupying lesions
- ③.patients with cancer and other serious underlying diseases
- ④.Patients with diffuse axonal injury
- ⑤.Incomplete clinical data and imaging data

All inpatients were treated strictly in accordance with clinical guidelines for neurosurgery, and all patients requiring surgical treatment were performed by experienced clinicians.

### Grouping

According to the GOS score of 3 months follow-up, the prognosis of the patients was divided into good group and bad group. GOS1–3 was defined as poor prognosis and 4–5 as good prognosis.

### Data extraction

#### Clinical data

Clinical data of patients were collected, including gender, age, ethnicity, GCS score at admission, time from injury to first CT, blood glucose level, Monocyte-to-Lymphocyte Ratio (MLR), C-G/L, complications (infection, epilepsy, thrombogenesis), intracranial pressure, and GOS score at 3 months. Among them, MLR and blood glucose information were extracted from the blood routine of patients within 24 h after admission, the glucose and lactic acid data of CSF were obtained through blood gas analyzer (GEM Premier 4000 system). Clinically, the degree of cerebral edema (CE) in patients with TBI usually reaches its peak 48–96 h after injury [4]. We routinely performed intracranial pressure monitoring for three consecutive days after admission to patients with traumatic brain injury to understand the changes in ICP and adjust medication in time. In addition, CSF biochemistry, routine CSF and CSF blood gas analysis of CSF samples are usually performed to monitor patients for the risk of intracranial infection and bacterial meningitis, especially for patients with open craniocerebral injury.

#### Imaging data

CT examinations were performed using a unified standard (axial thickness of 5 mm). The data provided by brain CT included Basal cistern status, Third ventricle status, MLS, CT grade of cerebral edema, contusion site, volume of contusion, mean CT value around contusion, and hematoma type (epidural hematoma, subdural hematoma). Among them, basal cistern status、third ventricle status、MLS、volume of contusion were all extracted from the first CT examination of patients admitted to hospital. Data on the grading of cerebral edema were extracted from CT data reviewed on the third day of admission. Images were viewed on the image viewing software, and all extracted data for each patient were performed by two senior clinicians and the areas of disagreement were reextracted until a final agreement was reached.

#### CT grading of CE

**Fig. 2:**
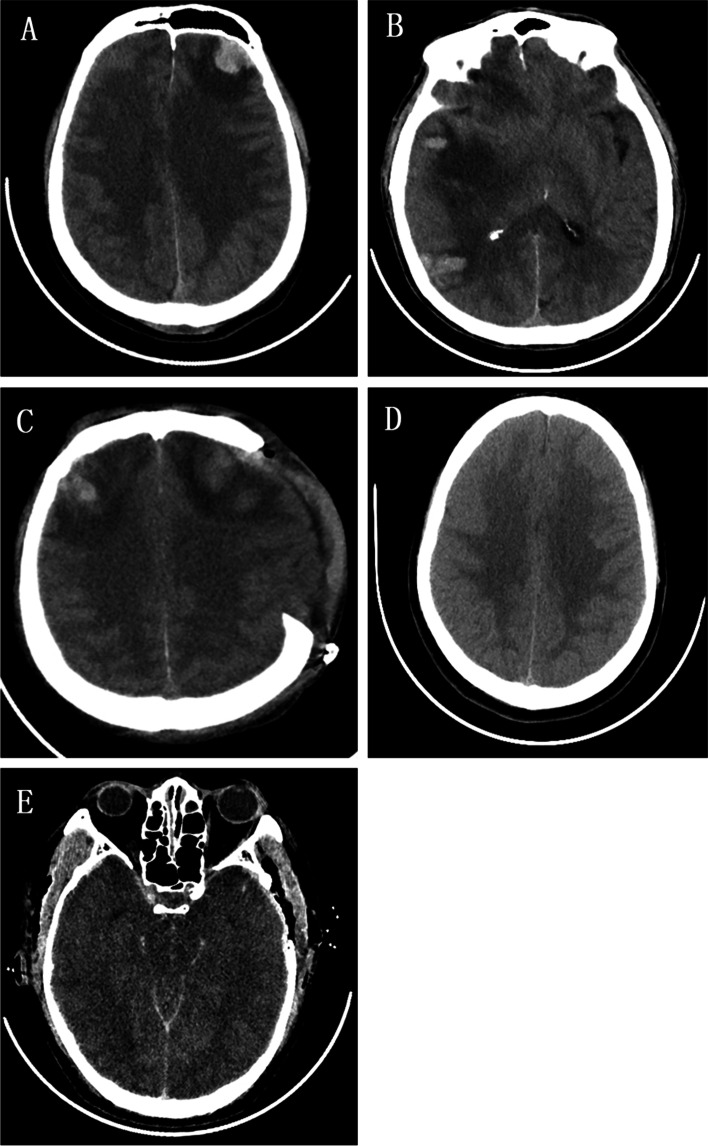
**A** Level 1:Focal CE limited to 1 lobe; **B** Level 2:Unilateral CE (> 1 lobe); **C** Level 3:Bilateral CE; **D** Level 4:Global CE (disappearance of sulcal relief); **E** Level 5:Global CE (disappearance of sulcal relief + completely effaced basal cisterns)

After TBI, CE reaches its peak 48–96 h after TBI [4], and decreases with the improvement of clinical symptoms and the decrease of ICP [5]. A CT grading method for CE was first presented in a recent report [6]. The specific grading is as follows:

A5(+ 1) point scale was developed (Fig. 2A~E):

- 0 No CE
- 1 Focal CE limited to 1 lobe
- 2 Unilateral CE (> 1 lobe)
- 3 Bilateral CE
- 4 Global CE (disappearance of sulcal relief)
- 5 Global CE (disappearance of sulcal relief+completely effaced Basal cistern status)

1additional point: mass-occupying lesion with surrounding edema represented in MLS of the septum pellucidum >0.5 cm (for grades 1–4).

criteria for the diagnosis of CE were hypodense areas in the white matter, typically with finger-like expansion, loss of grey/white matter differentiation, focal effacement of sulci, or a combination of these findings. The underlying pathophysiology (cytotoxicity and vasogenic edema) was not distinguished.

#### Measurement of volume of contusion and CT value around contusion

**Fig. 3:**
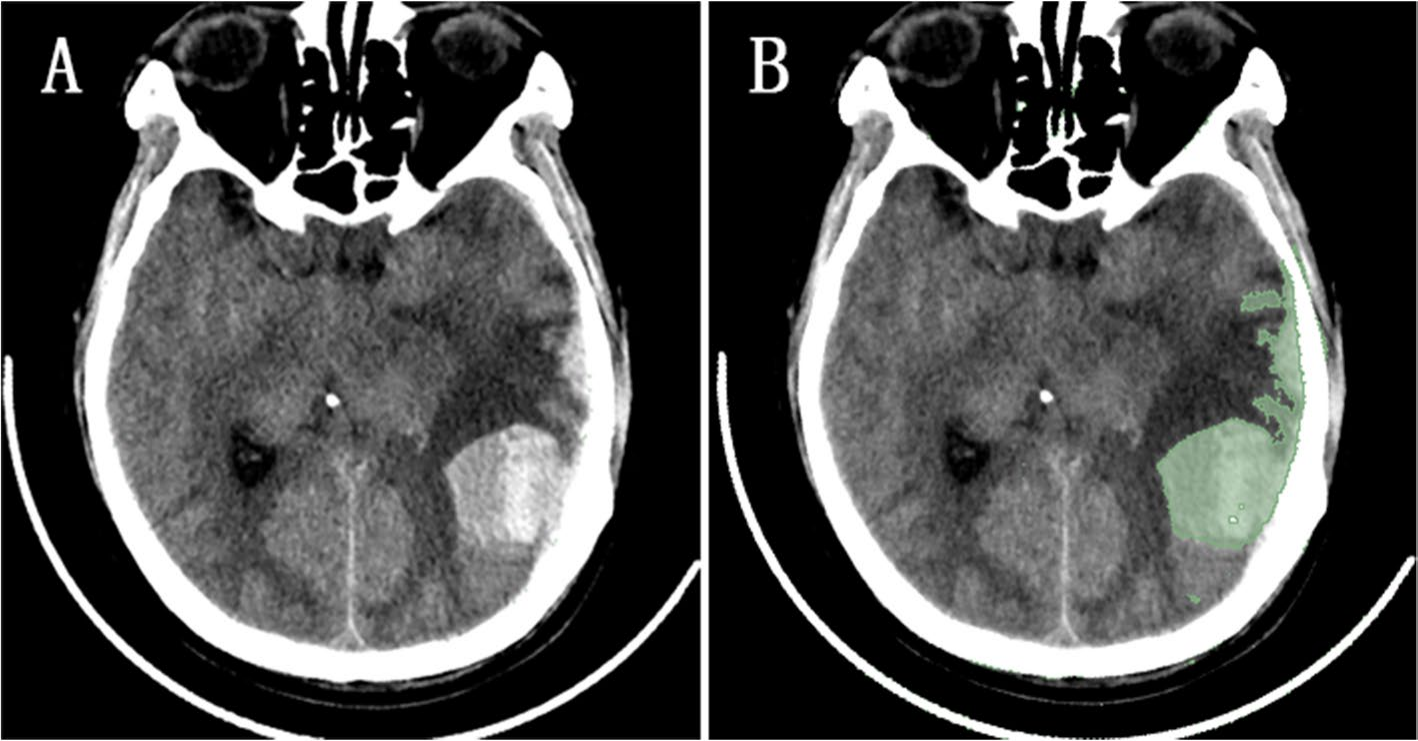
On 3D Slicer software, the Volume of contusion is provided by manually selecting the area of interest, setting thresholds based on Hounsfield units (fixed thresholds of 110 and 50 Hu) to distinguish contusions from the surrounding environment, and automatically summarizing adjacent voxels (**A** and **B**)

**Fig. 4:**
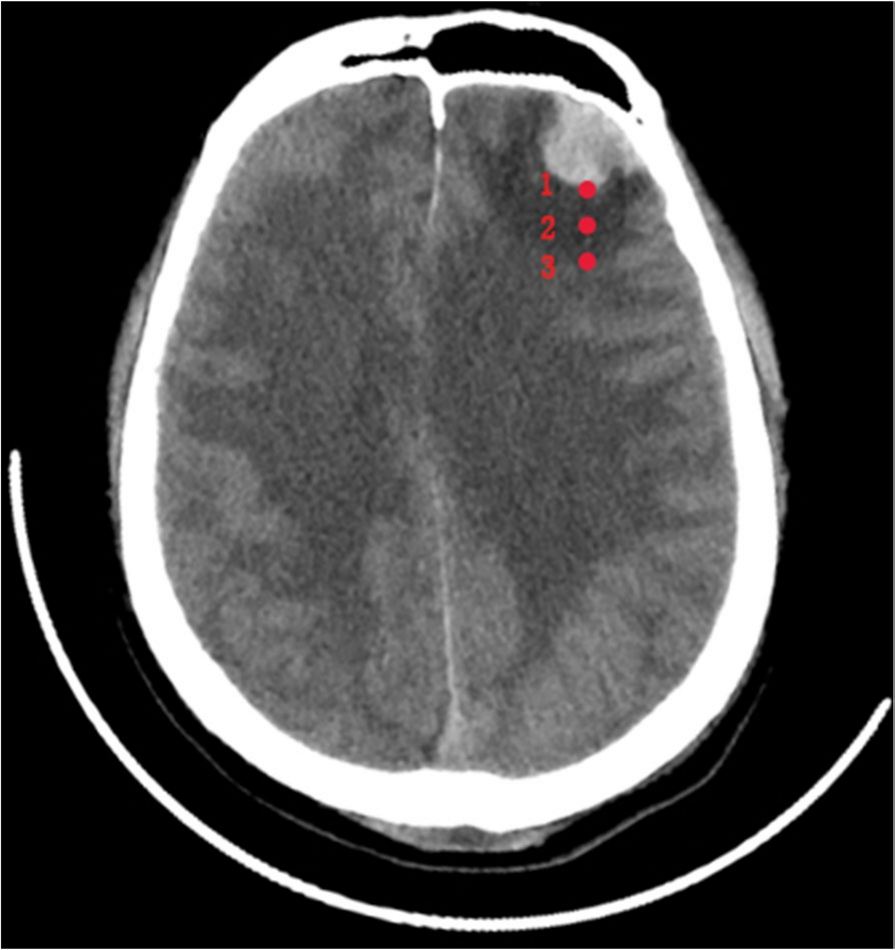
The maximum width of the edema zone around the contusion was selected, 3 points were equally spaced along the line and the corresponding CT values were recorded

The cranial CT data of patients within 24 h of admission were extracted, and the computer-aided software 3D Slicer (version 4.8.0;Harvard University, New York) is used to measure volume of contusion, as shown in Fig. 3 (A,B). The volume of contusion is provided by manually selecting the area of interest, setting thresholds based on Hounsfield units (fixed thresholds from 50 to 110Hu) to distinguish contusion from the surrounding environment, and automatically summarizing adjacent voxels. CT data of the head reexamined on the third day of admission were selected. The maximum width of the edema zone around the contusion was selected on the CT image reading software, and 3 points were equally spaced along this line, and the corresponding CT values were recorded and averaged (Fig. 4). For patients with multiple CC, the two largest areas of CC were selected for the same measurement method, and the average value was finally obtained.

#### Partitioning of data

In order to reflect the core value and rationality of modeling, we divided the collected continuous variable data into equal parts.

### Statistical analysis

Firstly, the collected data were randomly divided into the training group and the verification group according to 2:1, and R software was used for all analysis. Based on the training set data, LASSO regression was used to perform 10-fold cross validation to select clinical indicators with prognostic significance. Then, based on the results of regression analysis, a nomogram was drawn to predict the prognosis of patients with cerebral contusion. The area under the curve (AUC) value of the receiver operating characteristic (ROC) and the C index were used to evaluate the predictive power of the model. A calibration map was drawn to evaluate the accuracy of the prediction model, and a clinical decision curve was drawn to evaluate the patient benefit of the model. The model was internally validated using a validation group, and the model was also evaluated by drawing the ROC curve, calibration chart, clinical decision curve analysis (DCA), and calculating the C index. P<0.05 was considered statistically significant.

## Results

### Establishment of a prognostic model for CC

**Fig. 5:**
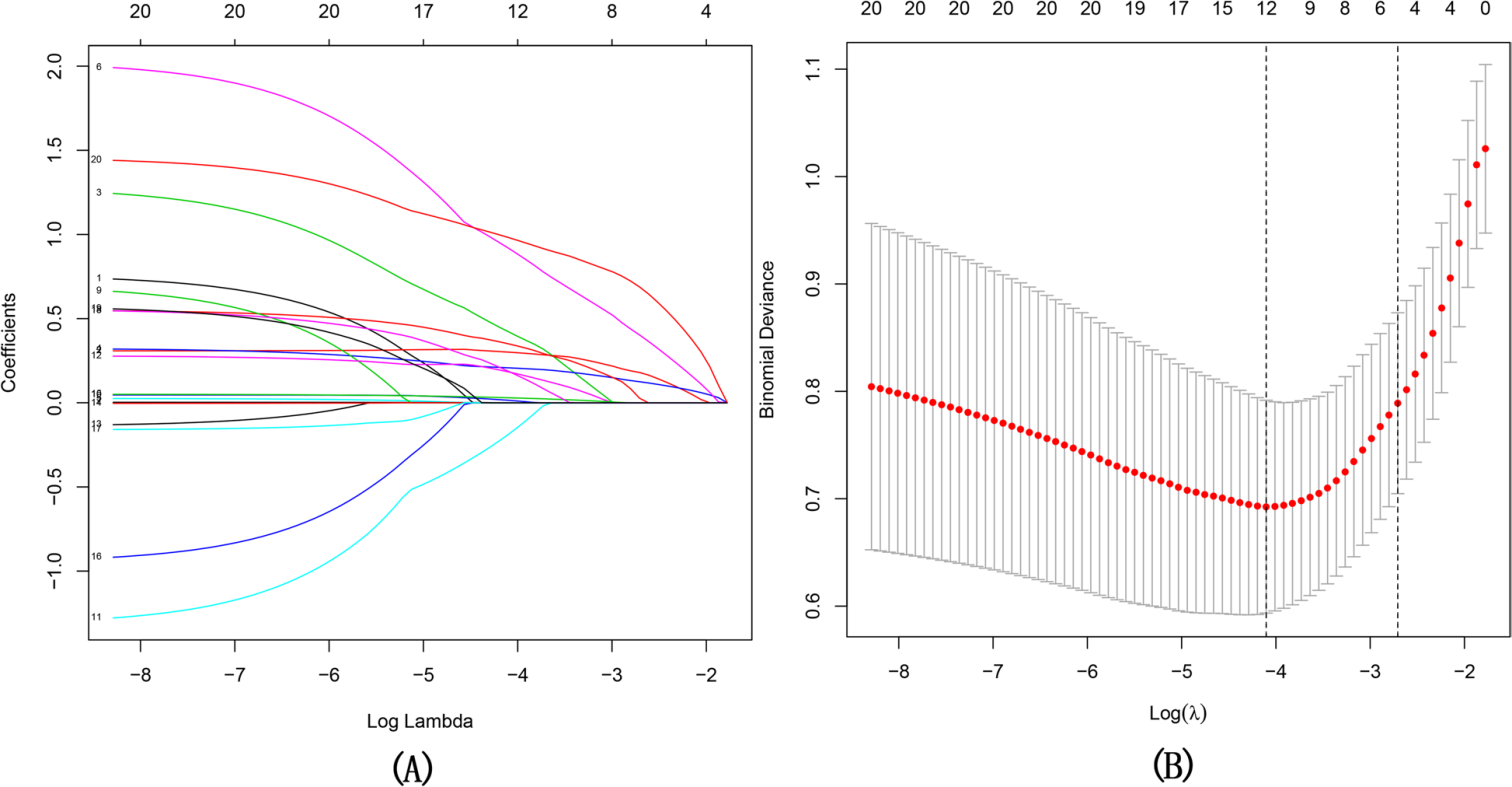
**A** LASSO coefficient profiles of the 20 texture features produced based on log (λ) sequence. 10-fold cross-validation was used for optimal λ resulting in 12 nonzero coefficients and vertical line drawn. **B** T uning parameter (λ) was selected by LASSO model using 10-fold cross-validation via minimum criteria. The vertical axis represents AUC which was plotted versus log(λ). The minimum criteria and the 1 standard error of the minimum criteria was chosen as the optimal values for dotted vertical lines drawn

**Fig. 6:**
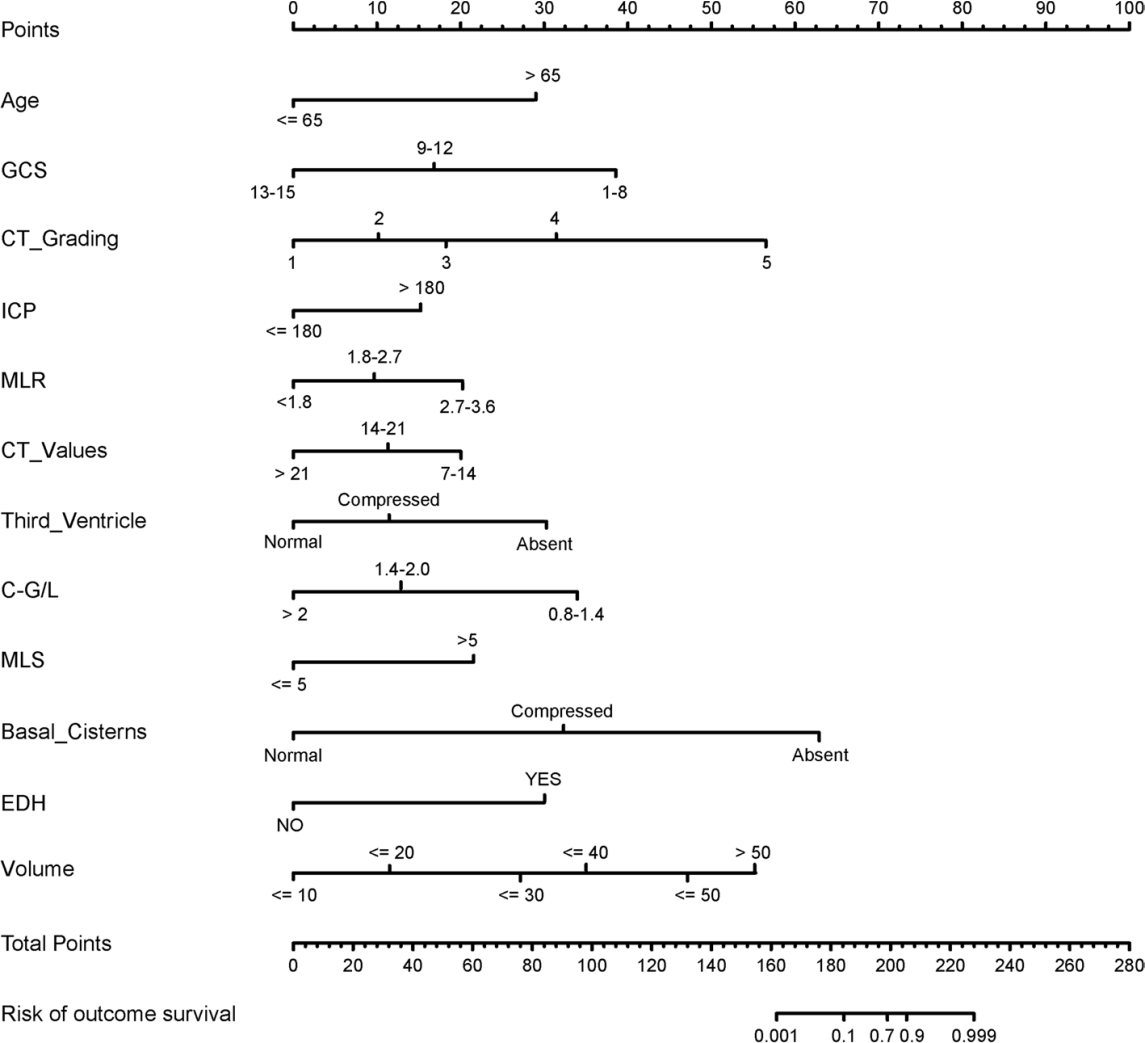
Logistic regression was carried out with software R to draw the nomogram

A total of 426 CC patients who met the inclusion criteria were enrolled in our study and were divided into a training group (*n* = 284) and a validation group (*n* = 142) (Fig. 1). No significant differences were found in general data between the training and validation groups (Table 1). All the included influencing factors were screened through Lasso logistic regression (Fig. 5A,B). Based on the regression analysis results, Nomogram were ploted (Fig. 6). The results indicated that the independent risk factors for the prognosis of patients with cerebral contusion were age, GCS, Basal cistern status, MLS, Third ventricle status, ICP, CT grade of BE, volume of contusion, mean CT value of edema zone, MLR,C-G/L, and EDH. Judging by the score, CT grading, Basal cistern status, volume of contusion and GCS score had a greater influence on the prognosis.

**Table 1:** Clinical characteristics of patients in the training and validation cohort

Variables	Training cohort (*N* = 284)	Validation cohort(*N* = 142)	I^2^	*P* Value
Age(Y)			3.468	0.672
<65	244	122		
≥65	40	20		
SEX			2.142	0.704
M	236	121		
F	48	21		
Basal cistern status			1.451	0.519
Normal	212	109		
Compressed	65	29		
Absent	7	4		
MLS (cm)			2.830	0.651
≤0.5	163	72		
>0.5	121	70		
GCS			2.327	0.821
Mild	129	66		
Moderate	143	70		
Severe	12	6		
CT grading			3.484	0.253
1	74	28		
2	50	27		
3	90	49		
4	50	31		
5	20	7		
ICP(mmH2O)			1.782	0.636
<180	60	40		
≥180	224	102		
C-G/L			3.328	0.485
0.8–1.4	80	33		
1.41–2.0	114	79		
>2.0	90	30		
CT Value(Hu)			1.238	0.173
7–14	21	15		
14.1–21	213	102		
>21	50	25		
Volume of contusion (ml)			2.632	0.739
≤10	95	36		
≤20	79	38		
≤30	53	43		
≤40	50	22		
≤50	4	1		
>50	3	2		

*Y* Year, *mm* millimeter, *ml* milliliter, *Hu* Hounsfield units

### Performance, validation, and clinical utility of nomogram

**Fig. 7:**
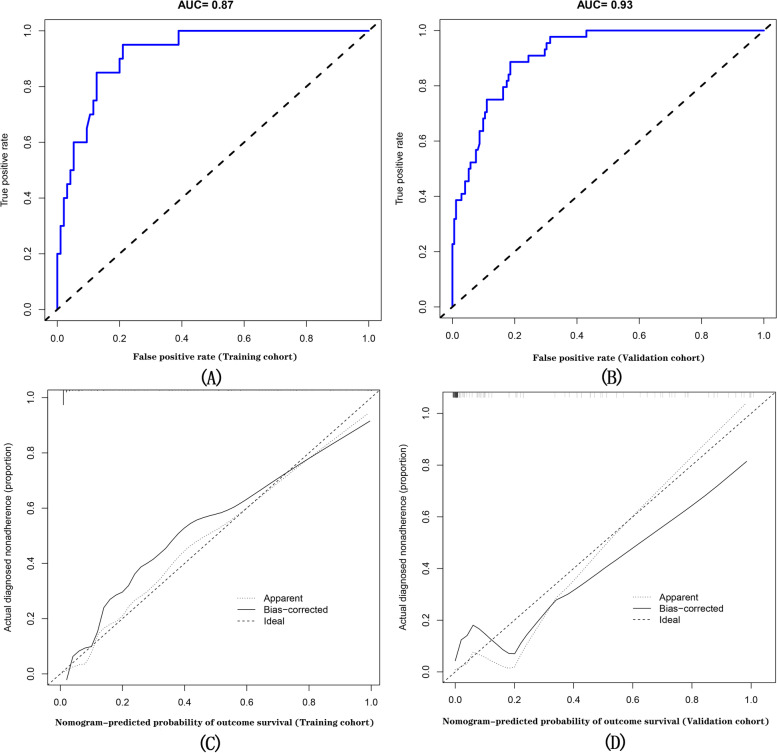
The ROC curve plotted showed that the AUC value of the training group was 0.87, and the AUC value of the validation group was 0.93, indicating the good prediction performance of the nomogram in the training and validation cohort (**A** and **B**. In internal and external validation of nomographic prediction of poor outcomes for CC, the calibration curves showed agreement between the observed outcomes for patients with CC (**C** and **D**). the dotted black line represents the predictive ability of the nomogram, a closer fit to the diagonal dotted black line represents a better prediction

**Fig. 8:**
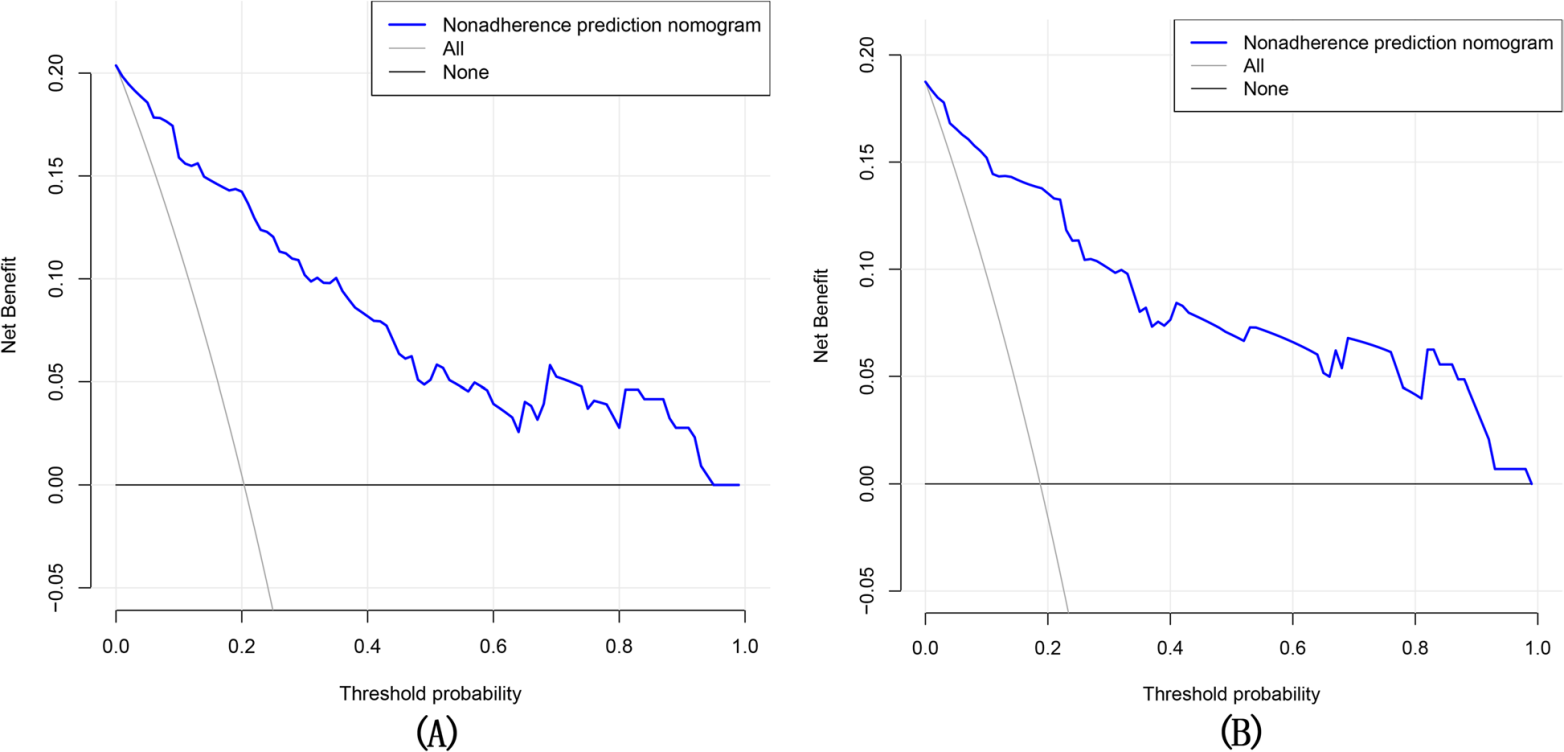
The clinical DCA of the training group (**A**) and validation group (**B**) was plotted using R software, and the results showed that the model brought A high benefit value to patients

The ROC curve showed that the AUC value of the training group was 0.87(95%CI, 0.026–0.952) (Fig. 7A), and the AUC value of the validation group was 0.93(95%CI, 0.032–0.965) (Fig. 7B). The nomogram showed good prediction performance. For this Nomogram calibration, the risk of a bad outcome is plotted on the X-axis;The actual probabilities are plotted on the Y axis, as shown in Fig. 7 (C, D), and the calibration diagram shows a good consistency between prediction and observation in the training cohort. In addition, to assess the clinical usefulness of the nomogram, the R software was used to plot the clinical DCA (Fig. 8A, B). The DCA provided insight into the range of predicted risks and the results showed that the model delivered a high value of benefit to the patient.

## Discussion

The most serious injury mechanism in TBI is hemorrhagic CC, which causes permanent damage to brain tissue. The severity of injury is related to the direct injury and secondary injury reaction caused by external force, such as CE, ICP elevation, microvascular disease, epileptic seizure, etc. Imaging and laboratory examination results of patients hide many prognostic indicators, which can provide important information for early, timely and accurate diagnosis of the disease. Therefore, in this study, high risk factors affecting the prognosis of patients were screened out by extracting baseline information, imaging and laboratory data. Age, GCS, Basal cistern status, MLS, Third ventricle status, ICP, CT grade of CE, volume of contusion, mean CT value of edema zone, MLR, C-G/L, EDH, provide important prognostic information for clinicians.

Our results are consistent with previous findings that low GCS score is highly associated with poor prognosis. However, GCS is an uncertain factor because it is affected by many factors, as the eye and speech parts of the GCS may be affected by drugs, sedatives, alcoholism, or endotracheal intubation, sometimes leading to inaccurate scoring [7]. In general, age is one of the independent risk factors for poor prognosis in many diseases. Our study found that age can significantly affect the prognosis of patients, possibly because the elderly have microvascular lesions and fragile blood vessel texture, which are more likely to develop progressive hemorrhagic injury after head trauma [8]. Degenerated and traumatized blood brain barrier (BBB) is also more permeable to post-traumatic inflammatory cells and factors, and therefore more likely to cause vasogenic and cytotoxic edema [9]. Oliveira et al. [10], through the monitoring of Transcranial color-coded duplex sonography on patients with traumatic brain injury, found that midline displacement, basal cistern and the compression degree of the third ventricle on brain CT scan were a strong predictor of intracranial hypertension. However, brain atrophy is more common in the elderly, which can provide more compensatory space for CE and intracranial hypertension,it will also make the MLS, compression of basal cistern and the third ventricle becomes not obvious, so just from the MLS, basal cistern status and the third ventricle degree to evaluate patients, may not be accurate enough in elderly patients.

Among the secondary lesions after CC, the most serious is uncontrollable brain swelling, which leads to decreased cerebral perfusion and further aggravation of hypoxic edema in brain tissue. In severe cases, it may cause mass effect or even fatal cerebral hernia. In clinical work, cranial CT is the preferred imaging examination method for patients with TBI, which can help us clarify the condition and provide prognostic information in the first time, so as to help us formulate treatment plans, which is especially beneficial in acute environments. Despite these advantages, the interpretation of CT images, especially in the detection of CE, has been questioned because it relies heavily on the radiologist’s subjective judgment of subtle differences in the attenuation of CE [11]. Recently, Lietke et al. [6] proposed the CT grading method for CE for the first time. According to the A5(+ 1) point scale, we extracted the head CT examination data on the third day of hospitalization and scored the edema on the head CT data of each patient. CT grading of cerebral edema is an independent predictor of patient prognosis. In the future, it is expected to contribute to more accurate diagnosis of the disease by clinicians. In addition, after craniocerebral injury, CE gradually worsened. Lietke et al. [6] found that the grade of BE was significantly correlated with ICP and prognosis through the statistics of continuous monitoring values of ICP on the third day after admission. In our study,although our results were significant, our results did not show a particularly good correlation with the original study. Probably because CE of acute period, clinical interventions tend to give priority to with dehydration drugs, within the scope of the security, Clinicians tend to increase the dose of dehydrating drugs as much as possible to reduce ICP. In addition, surgical decompression will also affect the intracranial pressure.

Some studies used computer-aided software 3D Slicer to accurately calculate the hematoma volume of hypertensive intracerebral hemorrhage and found that the hematoma volume could significantly affect the prognosis of patients with spontaneous cerebral hemorrhage [12–14]. Therefore, we also used 3D Slicer software to measure the contusion volume of patients with CC by selecting the region of interest and setting the HU (50–110) range, and included the measured results into the study after equal division at 10 ml interval. Our study found that the volume of contusion also significantly affected the prognosis of patients. The larger the volume of contusion, the worse the prognosis. Although the results we measured are obviously related to the prognosis of patients, there is a lack of such a tool to accurately measure the volume of contusion in clinical practice. Currently, Coniglobus formula is widely used in clinical practice to roughly measure the volume of intracerebral hemorrhage, which is convenient and fast, but with a large error. To our knowledge, this study is the first to accurately calculate the volume of contusion and use nomogram to evaluate the patient’s prognosis.

In general, the heavier the CE, the higher the water content of brain tissue, and the lower the CT value. Mangel et al. [15] found that the CT value of CE ranged from 0-28HU.Through the segmentation imaging protocol, Nguyen et al. [16] found that the average brain CT value of patients with extra-axial hematoma causing mass effect was closely related to the degree of brain tissue compression, and pointed out that CT HU value could be used as an auxiliary means to evaluate TBI. Kim et al. [17] found in their evaluation of a pediatric patient population with TBI that patients with CT values ranging from 17 to 24 Hu were highly associated with severe CE. In our previous study, it was found that the difference between preoperative and postoperative Gray-White Matter Ratio (GWR) could predict the prognosis of patients 3 months after surgery for extra-axial hematoma [18, 19]. Therefore, we measured the CT value of the edema zone around the contusion site to explore its value for the prognosis of patients with CC. Due to the relatively urgent situation of patients admitted to the emergency department, Many medical institutions also do not have the equipment such as CT segmentation imaging protocol, which can accurately measure the density of brain tissue, and the cost is high. We designed a convenient and quick rough measurement method, and selected the average CT values of 3 points in the edema zone as the average CT values of the edema zone and included them in the study. The measured CT values were trisected by an interval of 7, with the minimum of 7HU and the maximum of 26HU. The results showed that the lower the CT values of the edema around the CC, the worse the prognosis of patients. This can allow clinicians to have a general understanding of the patient’s edema level, which can help guide the next step of medical treatment.

MLR, as a cheap and readily available biomarker. Recently, multiple linear regression has been used to predict the prognosis of various diseases, including cancer [20], cardiovascular disease [21] and neurological disorders [22]. Neuroinflammation contributes to the pathology of CC and influences its process [23]. In patients with CC, monocytes, neutrophils and lymphocytes can invade from the periphery and participate in the injury response without the control of the BBB. Sheng et al. [24] established a prediction model of cerebral rebleeding risk after craniocerebral injury, and concluded that MLR had a great predictive value for the expansion of acute traumatic parenchymal hematoma. Therefore, this study included MLR as a variable in the study to explore its predictive value for the prognosis of patients with CC. We found that MLR was also an independent predictor of poor prognosis in patients with contusion. The larger the MLR, the worse the prognosis. This may be because monocytes play an important role in the secondary brain injury after CC, leading to increased bleeding and worsening edema of CC, which has a negative impact on recovery [25]. Low lymphocyte levels have also been found to be associated with spontaneous intracerebral parenchymal hematoma enlargement and clinical deterioration [26]. However, the specific role of lymphocytes in the acute stage of CC is still unclear, and the potential role of T lymphocytes in the secondary injury of patients with acute CC may be complicated due to the existence of a variety of cell subtypes.

Neurological outcomes in patients with TBI are largely influenced by secondary brain injury. Several studies using microdialysis techniques to assess cerebrospinal fluid glucose concentrations have found a weak correlation with systemic concentrations, confirming that glucose supply to brain tissues is independent of insulin but dependent on specific glucose transporters located in capillaries and the BBB [27–30]. After TBI, cerebral hypoxia due to microvascular abnormalities, increased ICP, cerebral hypoperfusion, or epileptic seizures may lead to decreased cerebral glucose levels. In addition, mitochondrial dysfunction may cause lactic accumulation and reduced clearance of lactic acid through the choroid plexus [31]. Therefore, monitoring CSF glucose and lactate concentrations may have potential prognostic value in CC patients. Cerebrospinal fluid glucose and lactate concentrations were measured with an arterial blood gas analyzer and c-G /L was calculated. Our results are consistent with expectations, and C-G/L may be valuable in detecting cerebral hypoxia episodes and predicting prognosis. In clinical practice, brain microdialysis is not widely used, and the analysis of glucose and lactic acid concentration in cerebrospinal fluid is easier to carry out.

Patients with CC combined with EDH are often accompanied by skull fracture, and the blood supply of the skull is rich, which is easy to cause EDH and cause space occupying effect in a short time. If the compression is removed in time by surgery, the treatment effect of the patients will be obvious [32]. In this study, CC sites were included, including frontal lobe, parietal lobe, temporal lobe and occipital lobe. Statistical results showed that the differences were not statistically significant(P>0.05), which was inconsistent with previous reports [24]. We analyzed the reasons as follows: Basal ganglia and brain stem or cerebellum is human body important vital center, once the damage prognosis is poor, but we are all included in patients with contusion parts above the tentorium, if not acute oppression brain stem hemorrhage or edema, generally will not endanger the patient lives, usually only can cause injury of brain tissue function is impaired, leaving related sequelae.

The incidence of poor prognosis in CC patients is closely related to contusion rebleeding, increased ICP and CE [33]. Malignant CE is particularly difficult to control because it is often insensitive to drug therapy and leads to irreversible damage, with a mortality rate of close to 100% in untreated patients. Of the 426 CC patients included in our study, 53 underwent decompressive craniectomy due to contusion rebleeding or increased CE. Postoperative surgical interventions (ICP monitoring, CSF drainage) and medical interventions (hypertonic therapy, sedation and analgesia, hypothermia) resulted in a reduction in ICP and mortality, but no recovery of neurological function once it was damaged.

## Conclusion

This study provides an easy-to-use tool for predicting prognosis and helps to optimize the classification and management of CC patients. Volume of contusion, CT value of edema band and C-G/L, which were introduced as variables for the first time in this study, were independent predictors of patient prognosis. However, since this study was a double-center retrospective study, a well-designed multi-center study with a large sample size is still needed to supplement and verify the results.

### Limitation

First of all, this is a retrospective, double institution study, which requires a multi-center, large-sample study for further demonstration. Secondly, this study did not distinguish surgical patients from non-surgical patients, and surgery itself may have an impact on the prognosis of patients. Thirdly, routine blood tests only provide overall indicators of monocytes and lymphocytes, without detailed analysis of monocytes and lymphocyte subsets.

## Supplementary Information

**Additional file 1.** Supplementary table of the results of the LASSO Regression Analysis with the Clinical Variables.

## Data Availability

The datasets used and/or analysed during the current study available from the corresponding author on reasonable request.

## Abbreviations

CC: Cerebral contusion
TBI: Traumatic brain injury
MLS: Midline shift
GCS: Glasgow coma score
ICP: Intracranial pressure
DCA: Decision curve analysis
CSF: Cerebrospinal fluid
C-G/L: CSF-Glucose/lactate ratio
MLR: Monocyte-to-lymphocyte ratio
CE: Cerebral edema
ROC: Receiver operating characteristic
AUC: Area under receiver operating characteristic curve
EDH: Epidural hematoma
SDH: Subdural hematoma
