# Supplementary material for: Establishment and validation of prognosis model for patients with cerebral contusion
Source: BMC Neurol. 2021 Nov 29;21:463. doi: 10.1186/s12883-021-02482-4 (PMC8628400; doi:10.1186/s12883-021-02482-4)
Supplement: Supplementary file 1 — Additional file 1. Supplementary table of the results of the LASSO Regression Analysis with the Clinical Variables. [file 12883_2021_2482_MOESM1_ESM.docx]

| Item | Coef |
| --- | --- |
| Age | 0.361658886 |
| GCS | 0.317492184 |
| CT_Grading | 0.359529051 |
| ICP | 0.039003104 |
| MLR | 0.068274571 |
| CT_values | 0.059694321 |
| Third_ventricle | 0.143223457 |
| Glucose_cerebrospinal | 0.173252056 |
| MLS | 0.060245291 |
| Basal_cisterns | 0.361658886 |
| EDH | 0.303113467 |
| Volume | 0.329489226 |

Table.Supplementary table of the results of the LASSO Regression Analysis with the Clinical Variables.
